# RNA expression of TLR10 in normal equine tissues

**DOI:** 10.1186/s13104-016-2161-9

**Published:** 2016-07-19

**Authors:** Rachael E. Tarlinton, Lauren Alder, Joanna Moreton, Grazieli Maboni, Richard D. Emes, Sabine Tötemeyer

**Affiliations:** School of Veterinary Medicine and Science, University of Nottingham, Sutton Bonington Campus, Loughborough, LE12 5RD UK; Advanced Data Analysis Centre, University of Nottingham, Sutton Bonington Campus, Loughborough, LE12 5RD UK

**Keywords:** TLR10, Toll-like receptor, Horse, *Equus caballus*, RNA

## Abstract

**Background:**

Toll like receptors are one of the major innate immune system pathogen recognition systems. There is little data on the expression of the TLR10 member of this family in the horse.

**Results:**

This paper describes the genetic structure of the Equine TLR10 gene and its RNA expression in a range of horse tissues. It describes the phylogenetic analysis of the Equine TLR1,6,10,2 annotations in the horse genome, firmly identifying them in their corresponding gene clades compared to other species and firmly placing the horse gene with other TLR10 genes from odd-toed ungulates. Additional 3’ transcript extensions to that annotated for TLR10 in the horse genome have been identified by analysis of RNAseq data. RNA expression of the equine TLR10 gene was highest in peripheral blood mononucleocytes and lymphoid tissue (lymph nodes and spleen), however some expression was detected in all tissues tested (jejunum, caudal mesenteric lymph nodes, bronchial lymph node, spleen, lung, colon, kidney and liver). Additional data on RNAseq expression of all equine TLR genes (1–4 and 6–10) demonstrate higher expression of TLR4 than other equine TLRs in all tissues.

**Conclusion:**

The equine TLR10 gene displays significant homology to other mammalian TLR10 genes and could be reasonably assumed to have similar fuctions. Its RNA level expression is higher in resting state PBMCs in horses than in other tissues.

**Electronic supplementary material:**

The online version of this article (doi:10.1186/s13104-016-2161-9) contains supplementary material, which is available to authorized users.

## Background

The toll-like receptors (TLRs) are a family of membrane receptors that recognise a wide range of pathogen associated molecular patterns (PAMPs) and are considered a crucial signaling component of the innate immune system. TLRs 1-13 have previously been identified in mammals [1]. These genes have a distinctive structure consisting of an extracellular amino-terminal leucine-rich repeat (LRR) domain, responsible for ligand binding, a transmembrane domain, and an intracellular carboxyl terminal toll/IL (interleukin)-1 receptor domain (TIR) that is responsible for signal transduction. Most TLR genes have no introns within their TIR domains. The genetic structure of these genes usually consists of a large 5′ exon containing the TIR and or the LRR domains with or without smaller exons at the 3′ end of the gene, often containing non-coding regions [2].

Based on genetic sequence TLRs can be broadly divided into six subfamilies that have related PAMP recognition and signaling pathways (TLR1/2/6/10, 3, 4, 5, 7/8/9 and 11/12/13). TLRs 3 and 7/8/9 are located intracellularly and respond to bacterial and viral nucleic acids, inducing expression of type I interferons. TLR 1/2/6/10 and 4, 5 are expressed on the cell surface and sense cell wall and outer membrane components of bacterial, fungal and protozoal organisms activating a core signaling pathway including NFκB and other transcription factors leading to pro-inflammatory cytokine release [3].

In the horse, the sequences of TLR 1–4 and 6–9 have been described [4–6] with some studies describing expression levels of some classes of TLRs in individual tissues or disease states [4, 5, 7–12]. The presence of equine TLR5 is controversial. Its existence has been suggested by cytokine release in response to bacterial flagellin, the ligand for TLR5 [10] and a recent report of a 454 bp fragment with high sequence similarity to the intracellular signaling domain of human TLR5 in an equine expressed sequence tag [13]. However, no full length homologue has been identified in the published equine genome.

TLR10 is currently the only orphan receptor without a known ligand, or conclusive biological function. A pseudogene in rodents [14], and full length TLR10 genes have been identified in a range of mammals, including humans [15], sheep [16], cattle [17] and pigs [18]. TLR10 is present as part of a 50 kb TLR6-TLR1-TLR10 gene cluster thought to have arisen by ancestral gene duplication events. These TLR1 subfamily proteins form heterodimers with TLR2 [3, 19] with varying ligand affinity.

TLR10 is known to be expressed in humans in a wide range of lymphoid tissues and cells, as well as in intestinal epithelial cells, dermal endothelial cells and placental trophoblasts [20–23]. TLR10 RNA expression has also been detected in pigs, cattle and sheep, primarily in lymphoid tissue such as peripheral blood leukocytes, lymph nodes and spleens, though trace levels of RNA have been detected in other tissues (Additional file 1: Table S1). Initial modeling studies have suggested that di and triacetylated lipopeptides may potentially interact with TLR10/1, TLR10/2 hetero-dimers and TLR10/10 homodimers [19]. Co-immunoprecipitation experiments confirmed that, similar to TLR2/1, TLR2/10 complexes recruit the proximal adaptor MyD88 to the activated receptor complex. However, human TLR10, alone or in cooperation with TLR2, fails to activate typical TLR-induced signalling (such as NF-kB activation) downstream of the adapter molecules [3]. The role of TLR10 in inflammation is controversial. TLR10 has been shown to mediate the inflammatory response in human intestinal epithelial cells and macrophages to intracellular *Listeria monocytogenes* infection in one study [24]. While another study, using RNA silencing, blocking antibody and transfection suggests inhibitory properties leading to an anti-inflammatory effect [25].

There has been only one published report on TLR10 expression in the horse. This report used semi-quantitative PCR, based on predicted sequences, and suggested TLR10 expression in equine bronchial epithelium [26]. To extend this understanding, this paper presents an initial description of the transcriptional profile of TLR10 in a variety of tissues in the horse, along with the previously described equine TLR genes.

## Methods

Tissue samples for RNAseq: details of these samples, generation of RNAseq data and mapping to the horse genome have been published previously [27, 28]. Briefly, the samples consisted of five tissue samples (kidney, jejunum, liver, spleen and mesenteric lymph node) collected from an aged gelding, lymphocytes isolated by Ficoll Paque (GE healthcare) from a healthy 11 year old welsh mountain pony gelding and RNA from lymphocytes isolated from a healthy thoroughbred mare (the same horse whose DNA the horse genome is derived from) (Additional file 1: Table S2). The RNA samples were extracted, prepared and sequenced on a SOLiD 3 ABi sequencer to generate 50 bp reads. These reads were mapped to the equine reference genome EquCab2 and the transcriptome data was generated as in Moreton et al. [27]. The data for the TLR genes was retrieved from the annotated transcriptome [data available at EBI sequence read archive (SRA) under the study accession number ERP001116 and at 10.7717/peerj.382/supp-6]. Relative expression is given as reads per kilobase per million reads (RPKM) [29].

Tissue samples for RT-qPCR based TLR10 expression analysis: Samples were collected post mortem from healthy animals euthanized for other reasons than this study. Approximately 5 mm^3^ tissue samples (kidney, spleen, liver, colon, lung, bronchial and mesenteric lymph nodes) were collected and stored in RNA later (Additional file 1: Table S2). Biopsies were homogenised using a 5 mm stainless steel ball-bearing in a Retsch^®^ Bead Mill MM 301 at 30 shakes per second for 4 min (colon, lung, bronchial and mesenteric lymph nodes), 5 min (spleen) or 6 min (liver and kidney), RNA extraction was performed using the Nucleospin RNA II mini kit (Machery Nagel) according to manufacturer’s instructions. RNA was converted to cDNA using random hexamer primers (promega) and M-MLV reverse transcriptase (promega) as per manufacturer’s instructions. RNA quality was determined using RNA 6000 Nano Kit^®^ Bioanalyser (Agilent technologies, Waldbronn, Germany), for RNA integrity numbers (RIN) see Additional file 1: Table S2. A cut off value of a RIN of 5 was set as the quality threshold for a sample to be included in the study as recommended for qPCR studies in Fleige and Pfaffl [30].

Tissues from all horses were considered to be in a “resting” state without obvious gross pathology.

For each predicted TLR like horse gene the longest open reading frame was translated and aligned with known TLR1,2,6 and 10 proteins from Swissprot. All known vertebrate TLR10 genes were identified and predicted protein sequences were identified. In each case, sequences were aligned using Muscle and a phylogenetic tree generated using PhyML under the LG model with 4 rate classes and NNI tree searching and 100 bootstrap replicates. The resulting trees were visualised using Figtree (http://tree.bio.ed.ac.uk/software/figtree/).

All quantitative reverse transcriptase real time PCR (RT-qPCR) experiments were designed and performed to comply with the quality controls detailed in the MIQE guidelines [31]. SYBR green RT-qPCR was performed on cDNA using primer sets for TLR10 and reference genes succinate dehydrogenase complex subunit A (SDHA) and Hypoxanthine phosphoribosyl transferase (HPRT) (Additional file 1: Table S3). Specificity of the PCR amplicons was confirmed by sequencing.

The Light Cycler 480 DNA SYBR green 1 master mix (Roche) in a 20 μl reaction volume was used according to manufacturer’s instructions. Cycling conditions consisted of 98 °C for 15 min then 45 cycles of 98 °C for 15 s, 58 °C for 30 s and 72 °C for 30 s. Reactions were performed on a Light Cycler 480 96 well plate real time PCR system (Roche). Serial dilutions of control cDNA were used to assess primer efficiency. Relative expression was calculated using the following formula [32]: Corrected Ct value = Ct + (Nt – Ct′) * S/S′ where Ct = mean sample Ct, Nt = experimental reference gene mean, Ct′ = mean reference gene of sample, S = TLR slope, S′ = reference gene slope.

## Results

Transcripts with (RPKM > 0) were identified for TLR1-4 and TLR6-9 as well as TLR10, the latter has not been reported previously. The Ensembl *Equus caballus* database does not contain any annotations for TLR5. By comparison to known TLR1,6 and 10 protein sequences, two horse TLR1 transcripts were identified (ENSCAT00000002340 and rmCuff_TCONS_00039270) and single transcripts for TLR6 (ENSCAT00000001932) and TLR10 (ENSCAT00000022185).

**Fig. 1:**
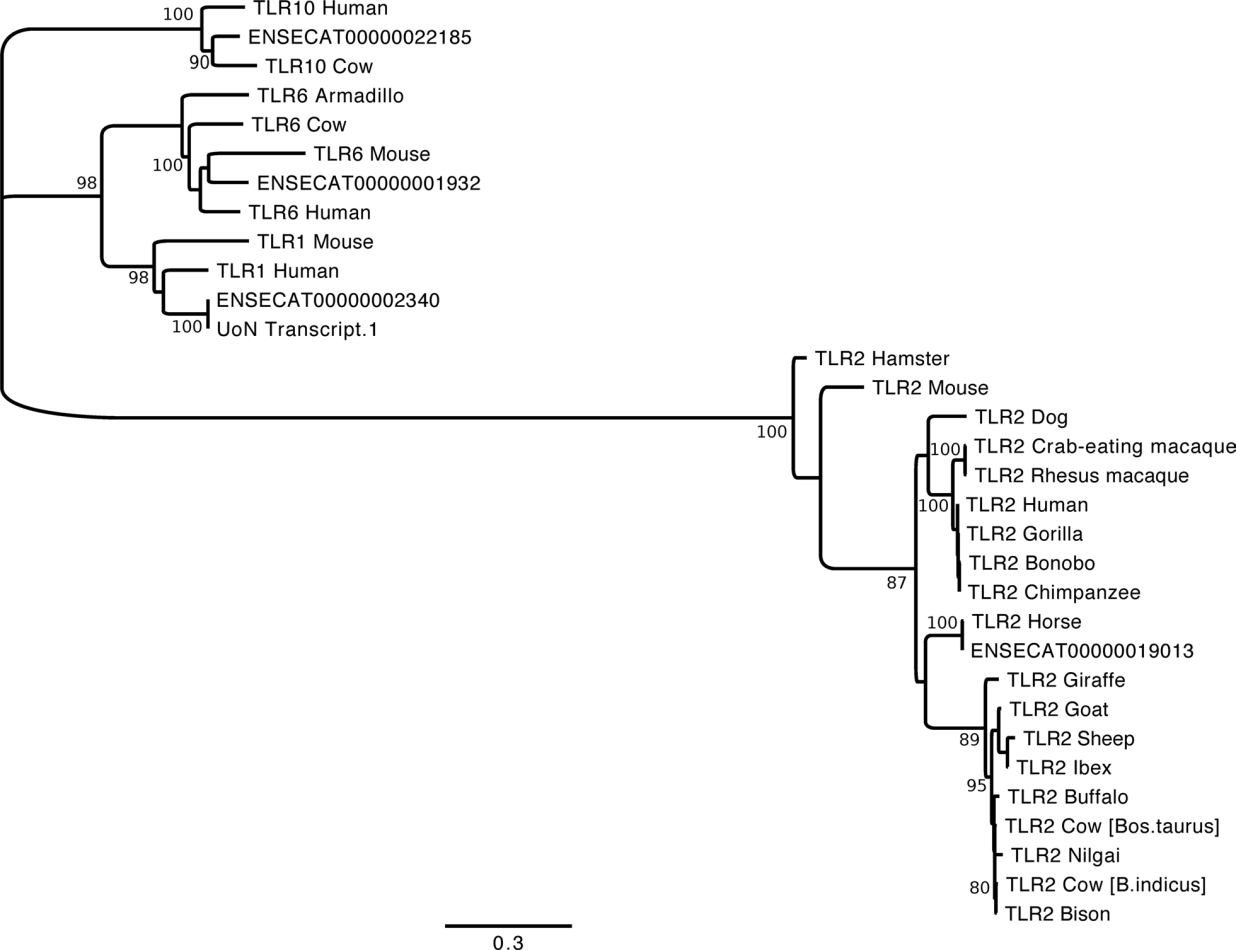
Phylogenetic tree of *TLR1,2 6,10* gene family. Comparison of the horse TLR transcripts identified in this study with known TLR1,2,6 and 10 protein sequences. Accession numbers of sequences are contained in Additional file 1: Table S4. Sequences were aligned using Muscle and an unrooted phylogenetic tree generated using PhyML (100 bootstrap replicates). For clarity, only bootstrap values above 80 % are shown

**Fig. 2:**
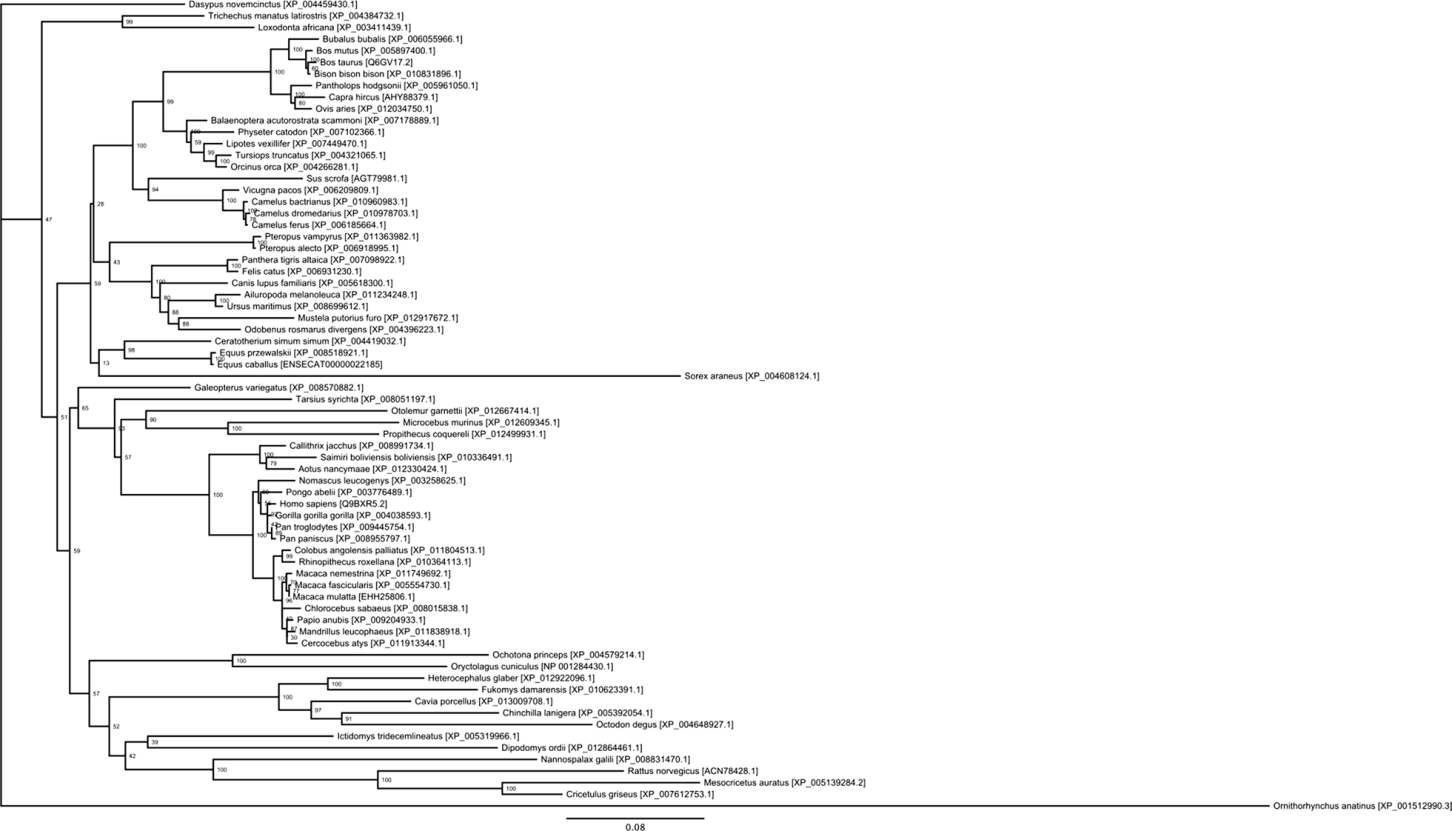
Phylogenetic tree of vertebrate TLR10 genes: Sequences were aligned using muscle and an unrooted phylogenetic tree generated using PhyML (100 bootstrap replicates). Bootstrap values are shown for each node. Species are identified by latin name and accession number. Details of sequences are contained in Additional file 1: Table S5

Comparison of Equine transcripts to representative TLR1,2,6 and 10 genes shows the clear definition of ENSECAT00000002340 as equine TLR1, ENSECAT00000019013 as TLR2, ENSECAT00000001932 as TLR6 and ENSECAT00000022185 as TLR10 (Fig. 1). When TLR10 transcript ENSECAT00000022185 is aligned with representatives of vertebrate TLR10 sequences it is most closely related to the wild Przewalski horse (*E. ferus przewalskii*) and the southern white rhinoceros (*Ceratotherium simum simum*) and all odd-toes ungulates (Perissodactyla) (Fig. 2) as would be expected.

**Fig. 3:**
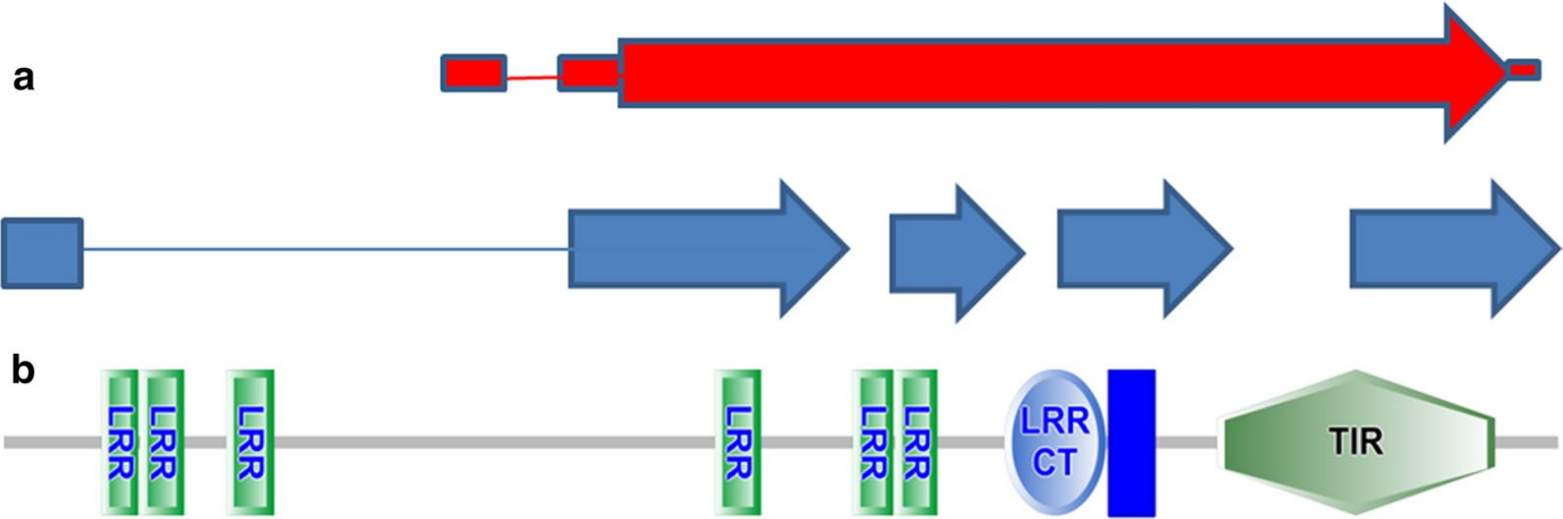
Schematic diagram of equine TLR10 transcript and protein structure. **a** eqTLR10 transcript structure: *Red*  Ensembl annotation (EquCab2), *Blue* predicted transcript structure from this study, *lines* predicted introns, *boxes and arrows* predicted exons, *arrows* indicate direction of transcription. **b** Predicted Protein domain architecture equine TLR10. Domain diagram generated using SMART (http://smart.embl-heidelberg.de/). *LRR* leucine rich repeat, *LRRCT* leucine rich repeat C-terminal domain, *Blue line* transmembrane domain, *TIR* carboxyl terminal Toll/IL (interleukin)-1 receptor domain

Sufficient read depth was available for transcript structure analysis for TLR10 (Fig. 3a). Gaps in the predicted transcripts compared with the ENSEMBL annotations over the 5′ exons are probably due to sub-optimal read coverage rather than real transcriptional variation. Additional 3′ transcript extensions are suggested by our transcript analysis when compared with the reference sequence annotations (Fig. 3a). The sequence covered by our predicted TLR10 transcript contains one large open reading frame with a short leader sequence. Basic structural domain analysis of this open reading frame using the SMART protein domain analysis suite demonstrated a typical mammalian TLR10 gene structure (Fig. 3b) consisting of a series of amino terminal LRR’s, a Leucine Rich Repeat C—terminal domain, a transmembrane domain and a carboxyl terminal TIR domain. Read depth was not consistently high enough for confident calling of SNPs on TLR10.

Each organ or cell type was found to have a distinct TLR expression profile (Additional file 2: Figure S1). Absolute TLR expression was highest in the lymphocyte samples (Additional file 2: Figure S1 A and B). Both lymphocyte samples had similar patterns of TLR expression with TLR4 expressed at the highest level (26 < RPKM < 70) followed by TLR2 and TLR1. TLR’s 6, 7, 8 and 10 were expressed at low levels (RPKM < 12) and TLR3 at trace levels (RPKM < 1). Apart from expression of TLR4 and 8 in the spleen, expression of any TLR in other tissues was at low or trace levels (RPKM < 5). Total number of reads and reads mapped per tissue were similar (Additional file 1: Table S2), indicating that the differences in RPKM reads for this class of gene is genuine and not due to differences in general read mapping density across tissues.

**Fig. 4:**
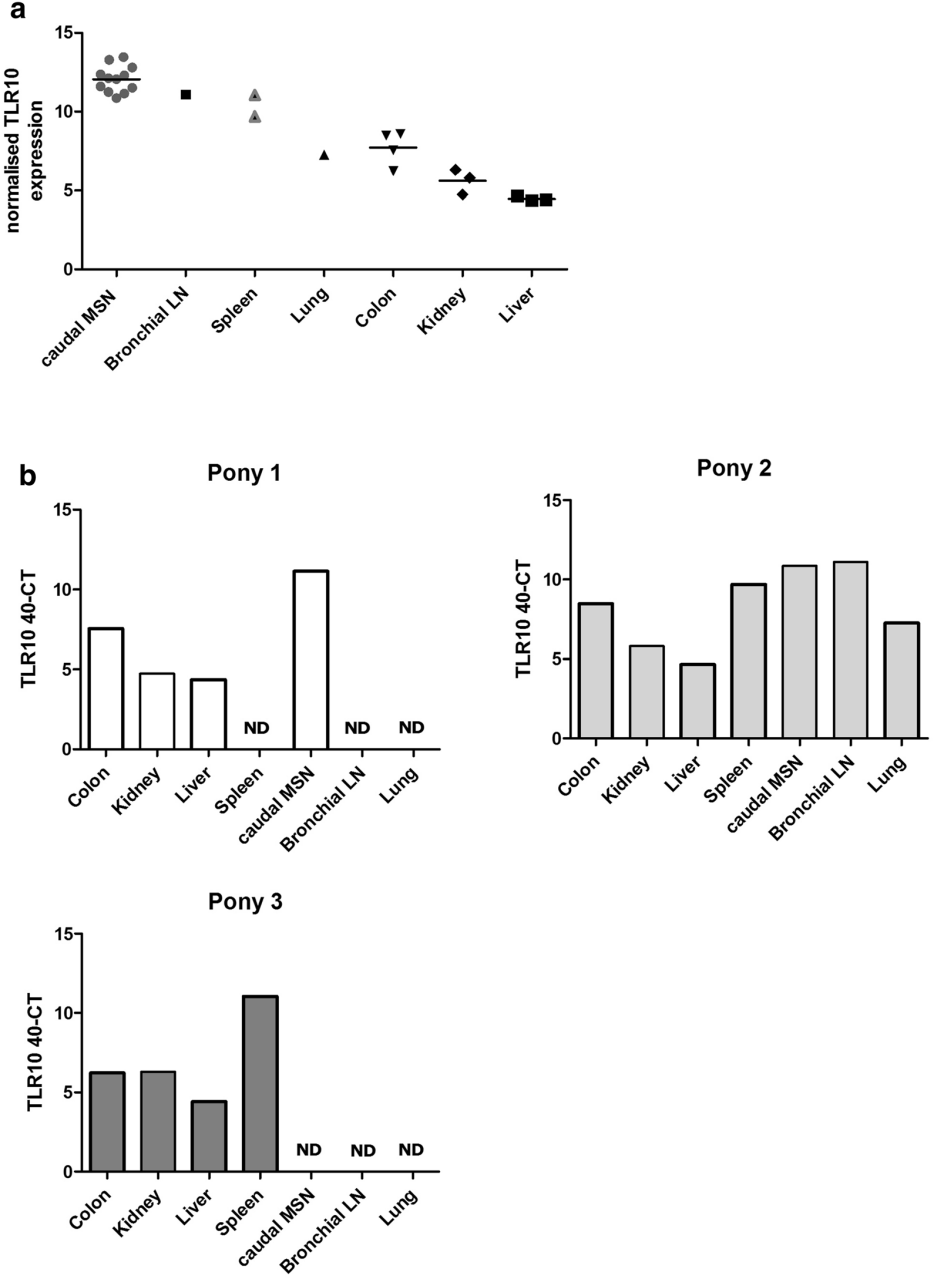
TLR10 expression in healthy equine tissues. **a** TLR10 expression determined by RT-qPCR. Samples from caudal mesenteric lymph nodes (MSN, n = 12), Bronchial lymph node (LN, n = 1), spleen (n = 2), lung (n = 1), colon (n = 4), kidney (n = 3) and liver (n = 3) were collected post slaughter/euthanasia and preserved in RNALater prior to RNA isolation, cDNA synthesis and RT-qPCR. TLR10 expression has been normalized to two house keeping genes, SDHA and HPRT and is expressed as 40-CT, mean is shown as *horizontal line*. ND = not determined (tissue not available) **b** TLR10 expression across different tissues in 3 individual horses where multiple tissue samples were available (these horses are also included in part A)

RNAseq data for TLR10 expression were very low in tissues other than PBMCs (Additional file 2: Figure S1). To confirm TLR10 mRNA expression from the RNAseq data, a range of tissues from further Thoroughbred Horses and Welsh Ponies were analyzed by RT-qPCR. TLR10 transcript was detected in all tissues tested: caudal mesenteric lymph nodes, bronchial lymph node, spleen, lung, colon, kidney and liver; with highest levels in the lymphoid tissues (lymph nodes and spleen) and lowest in liver (Fig. 4). Where samples for multiple horses were available for that tissue, TLR10 transcript levels were consistent within that tissue (Fig. 4a).

## Discussion

In summary, equine TLR10 shows conserved domain architecture and high sequence similarity to its mammalian counterparts. Equine TLR10 mRNA is expressed in a wide range of tissues with highest levels in lymphoid tissues.

The similarity of the predicted Equine TLR10 protein with the other described mammalian TLR10’s [33]) implies functional conservation between the equine and other TLR10’s. Our transcriptome assembly and annotation would suggest additional 3′ exons to the reference genome annotation for equine TLR10. These extensions may be tissue specific and remain to be confirmed with Sanger sequencing of mRNA.

The transcription profile for the complete TLR repertoire in the normal horse tissues examined here would also additionally suggest that TLR4 is an important sentinel signaling molecule in horse lymphoid tissue (spleen and PBMCs). This would correlate with the known susceptibility of horses to bacterial LPS (the ligand for TLR4) [34].

## Conclusion

The results presented here demonstrate transcription of TLR10 in the horse in a wide range of tissues with expression location and levels similar to that in other mammalian species (Additional file 1: Table S1). This paper provides a starting point for further studies of activities of this gene in the horse. Presumably given it’s homology with TLR10 in other species it is likely to form heterodimers with TLR2, however confirmation of the function of TLR10 in any mammalian species will require further work.

## Additional files

10.1186/s13104-016-2161-9 Additional tables.
10.1186/s13104-016-2161-9 TLR 1-4 and 6-10 RNA expression in horse tissues. Peripheral blood mononucleolyte cells (PBMC) from two horses (A–B) and tissues (Kidney (C), Liver (D), Mesenteric Lymph Node (E), Spleen (F) and Jejunum (G)) from a third. Y axis = Reads per kilobase per million base pairs (RPKM), X axis = TLR gene.

## Abbreviations

TLR: toll like receptor
PBMC: peripheral blood mononucleocyte cells
RT-qPCR: reverse transcriptase quantitative polymerase chain reaction
SDHA: succinate dehydrogenase complex subunit A
HPRT: hypoxanthine phosphoribosyl transferase
