# Supplementary material for: RNA expression of TLR10 in normal equine tissues
Source: BMC Res Notes. 2016 Jul 19;9:353. doi: 10.1186/s13104-016-2161-9 (PMC4952062; doi:10.1186/s13104-016-2161-9)
Supplement: Supplementary file 1 — 10.1186/s13104-016-2161-9 Additional tables. [file 13104_2016_2161_MOESM1_ESM.docx]

**Supplementary Data**

**Supplementary Table 1: TLR 10 expression in mammalian tissues**

| **Organs/cells** | **Equine**  **This study** | **Human** | **Porcine ^4^** | **Bovine^5^** | **Ovine^14^** |
| --- | --- | --- | --- | --- | --- |
| **Peripheral blood leukocytes** | ++^$^ | +^8,9^ |  | ++ | ++ |
| **Spleen** | ++ | +^2,10^ | ++ | + |  |
| **Lymph node** | ++ | +^2,10^ |  | ++ | ++ |
| **Small intestine/jejunum** | +^$^ | +^12^ | + |  |  |
| **Colon** | + |  | (+) |  |  |
| **Kidney** | + |  | (+) |  |  |
| **Lung** | + |  | + | (+) |  |
| **liver** | (+) |  | (+) | (+) |  |

**ND – not detected, (+++ strong, ++ medium, +obvious, (+) traces)**

**^$^ by RNA Seq;**

References:

^2^ Hasan 2005, ^4^ Shinkai 2006 ; ^5^Opsal 2006; ^8^Oosting et al 2014; ^9^Requena et al 2013; ^10^Chuang & Ulevitch 2001; ^11^Mulla et al 2013; ^12^Regan et al 2013; ^14^Chang et al 2009

**Supplementary Table 2: Equine tissues and their RNA quality**

| **Animal** | **source** | **tissue** | **Raw reads** | **Reads mapped** |
| --- | --- | --- | --- | --- |
| **RNA Seq** |  |  |  |  |
| Thoroughbred  (female) | D Miller | PBMC A | 20,853,992 | 9,490,429 |
| Welsh mountain pony (gelding) | J Kydd | PBMC B | 32,050,093 | 13,877,541 |
| Aged Gelding (osteoarthritis) |  | Jejunum  Kidney  Liver  Lymph node  Spleen | 19,902,170  33,158,285  23,176,545  24,671,029  30,421,675 | 9,064,806  12,875,162  10,237,855  10,998,429  15,185,938 |
| **qPCR** |  |  |  | **RIN** |
| Thoroughbred 1 | Abattoir | Caudal MLN |  | 9.4 |
| Thoroughbred 2 | Abattoir | Caudal MLN |  | 6.8 |
| Thoroughbred 3 | Abattoir | Caudal MLN |  | 9.1 |
| Thoroughbred 4 | Abattoir | Caudal MLN |  | 9 |
| Thoroughbred 5 | Abattoir | Caudal MLN |  | 7.7 |
| Thoroughbred 6 | Abattoir | Caudal MLN |  | 7.8 |
| Thoroughbred 7 | Abattoir | Caudal MLN |  | 8.1 |
| Thoroughbred 8 | Abattoir | Caudal MLN |  | 5.3 |
| Thoroughbred 9 | Abattoir | Caudal MLN |  | 9.2 |
| Thoroughbred 10 | Abattoir | Caudal MLN |  | 8.1 |
| Thoroughbred 11 | Abattoir | Colon |  | 9 |

| Welsh Mountain  Pony 1  (Gelding  16-18 months) | AHT | Caudal Mesenteric Lymph Node |  | 9.4 |
| --- | --- | --- | --- | --- |
|  |  | Kidney |  | 8.7 |
|  |  | Liver |  | 8.4 |
|  |  | Colon |  | 7.1 |
| Welsh Mountain  Pony 2  (Filly 16-18 months) | AHT | Caudal Mesenteric Lymph Node |  | 9.6 |
|  |  | Brochial Lymph Node |  | 9.2 |
|  |  | Kidney |  | 6.9 |
|  |  | Liver |  | 7.8 |
|  |  | Spleen |  | 9.2 |
|  |  | Colon |  | 7.9 |
|  |  | Lung |  | 8.2 |
| Welsh Mountain  Pony 3  (Gelding  16-18 months) | AHT | Kidney |  | 6.3 |
|  |  | Liver |  | 8 |
|  |  | Spleen |  | 7.7 |
|  |  | Colon |  | 7.9 |

RIN: RNA integrity number, AHT Animal Health Trust, MLN mesenteric lymph node, PBMC Peripheral blood monocytes

**Supplementary Table 3: Primers used in Q-PCR confirmation of TLR gene expression**

| **Gene** | **Primers** | **Reference** | **Efficiency (%)** |
| --- | --- | --- | --- |
| TLR10 | TGACAACCAACTGCTCCAAT  TATCAGATGCACAAATGCCA | This study  (amplicon: 81bp) | 102.7 |
| HPRT | AATTATGGACAGGACTGAACGG  ATAATCCAGCAGGTCAGCAAAG | Cappelli et al 2008 | 101.6 |
| SDHA | GAGGAATGGTCTGGAATACTG  GCCTCTGCTCCATAAATCG | Cappelli et al 2008 | 99.6 |

**Supplementary Table 4: Accession numbers of the sequences used in generation of Figure 1.**

| **Name** | **Organism of origin** | **SwissProt Accession number** |
| --- | --- | --- |
| TLR2 Human | *Homo sapiens*  Human | [O60603](http://www.uniprot.org/uniprot/O60603) |
| TLR2 Cow [b.indicus] | *Bos Indicus*  Zebu Cattle | B5T267 |
| TLR2 Cow [Bos.taurus] | *Bos Taurus*  Cattle | [Q95LA9](http://www.uniprot.org/uniprot/Q95LA9) |
| TLR2 Hampster | *Critelus griseus*  Chinese Hampster | [Q9R1F8](http://www.uniprot.org/uniprot/Q9R1F8) |
| TLR2 Sheep | *Ovies aries*  Domestic Sheep | [B2LT65](http://www.uniprot.org/uniprot/B2LT65) |
| TLR2 Bison | *Bison bison*  American Bison | B2LT61 |
| TLR2 Nilgai | *Boselaphys tragocamelus* Nilgai Antelope | [Q2V897](http://www.uniprot.org/uniprot/Q2V897) |
| TLR2 Buffalo | *Bubalus bubalis* Domestic water buffalo | Q2PZH4 |
| TLR2 Dog | *Canis familiaris*  Domestic Dog | Q689D1 |
| TLR2 Goat | *Capra hircus*  Goat | I0CCB6 |
| TLR2 Ibex | *Capra ibex*  Ibex | [B2LT62](http://www.uniprot.org/uniprot/B2LT62) |
| TLR2 Giraffe | *Giraffe Camelopardalis*  Giraffe | B2LT64 |
| TLR2 Gorilla | *Gorilla gorilla* Western Lowland Gorilla | [B3Y615](http://www.uniprot.org/uniprot/B3Y615) |
| TLR2 Chimpanzee | *Pan troglodytes*  Chimpanzee | [B3Y613](http://www.uniprot.org/uniprot/B3Y613) |
| TLR2 Horse | *Equus caballus*  Horse | Q6T752 |
| TLR2 Crab-eating macaque | *Macaca fasicularis* Crab eating macaque, Cynomologous monkey | Q95M53 |
| TLR2 Rhesus macaque | *Macaca Mulatta*  Rhesus macaque | [B3Y618](http://www.uniprot.org/uniprot/B3Y618) |
| TLR2 Bonobo | *Pan paniscus* Bonobo | B3Y614 |
| TLR2 Mouse | *Mus Musculus*  House mouse | Q9QUN7 |
| ENSECAT00000019013 | *Equus caballus*  Horse | Ensembl Horse genome annotation TLR2 |
| ENSECAT00000022185 | *Equus caballus*  Horse | Ensembl Horse genome annotation TLR10 |
| TLR6 Human | *Homo Sapiens*  Human | [Q9Y2C9](http://www.uniprot.org/uniprot/Q9Y2C9) |
| TLR6 Armadillo | *Dasypus novemcinctus*  Nine banded armadillo | Q0ZUL9 |
| TLR6 Cow | *Bos Taurus*  Cattle | [Q704V6](http://www.uniprot.org/uniprot/Q704V6) |
| TLR6 Mouse | *Mus Musculus*  House mouse | Q9EPW9 |
| ENSECAT00000001932 | *Equus caballus*  Horse | Ensembl Horse genome annotation TLR6 |
| ENSECAT00000002340 | *Equus caballus*  Horse | Ensembl Horse genome annotation TLR1 |
| UoN transcript 1 | *Equus caballus*  Horse | Horse Genome annotation Moreton et al 2014 [10.7717/peerj.382/supp-6](https://dx.doi.org/10.7717/peerj.382/supp-6) rmCuff_TCONS_00039270 |
| TLR10 Human | *Homo Sapiens*  Human | [Q9BXR5](http://www.uniprot.org/uniprot/Q9BXR5) |
| TLR10 Bovine | *Bos Taurus*  Cattle | Q6GV17 |
| TLR1 Human | *Homo Sapiens*  Human | [Q15399](http://www.uniprot.org/uniprot/Q15399) |
| TLR1 Mouse | *Mus Musculus*  House mouse | Q9EPQ1 |

**Suplementary Table 5: Accession numbers of the TLR10 sequences used in generation of Figure 2.**

| **Name of organism** | **GenBank accession number** |
| --- | --- |
| *Ailuropoda melanoleuca*  Giant Panda | XP_011234248.1 |
| *Aotus nancymaae*  Nancy Ma’s Night Monkey | XP_012330424.1 |
| *Balaenoptera acutorostrata scammoni*  Minke Whale | XP_007178889.1 |
| *Bison bison bison*  American Plains Bison | XP_010831896.1 |
| *Bos mutus*  Wild Yak | XP_005897400.1 |
| *Bos Taurus*  Cow | Q6GV17.2 |
| *Bubalus bubalis*  Asian Water Buffalo | XP_006055966.1 |
| *Callithrix jacchus*  Common Marmoset | XP_008991734.1 |
| *Camelus bactrianus*  Bactrian Camel | XP_010960983.1 |
| *Camelus dromedaries*  Dromedary Camel | XP_010978703.1 |
| *Camelus ferus*  Wild Bactrian Camel | XP_006185664.1 |
| *Canis lupus familiaris*  Domestic Dog | XP_005618300.1 |
| *Capra hircus*  Goat | AHY88379.1 |
| *Cavia porcellus*  Guinea Pig | XP_013009708.1 |
| *Ceratotherium simum simum*  Northern White Rhinoceros | XP_004419032.1 |
| *Cercocebus atys*  Sooty Mangabey | XP_011913344.1 |
| *Chinchilla lanigera*  Long Tailed Chincilla | XP_005392054.1 |
| *Chlorocebus sabaeus*  African Green Monkey | XP_008015838.1 |
| *Colobus angolensis palliates*  Angolan Colobus | XP_011804513.1 |
| *Cricetulus griseus*  Chinese Hampster | XP_007612753.1 |
| *Dasypus novemcinctus*  Nine Banded Armadillo | [XP_004459430.1] |
| *Dipodomys ordii*  Ord’s Kangaroo Rat | XP_012864461.1 |
| *Equus caballus*  Domestic Horse | ENSECAT00000022185 |
| *Equus przewalskii*  Przewalskii’s horse | XP_008518921.1 |
| *Felis catus*  Domestic Cat | XP_006931230.1 |
| *Fukomys damarensis*  Damaraland Mole Rate | XP_010623391.1 |
| *Galeopterus variegatus*  Sunda Flying Lemur | XP_008570882.1 |
| *Gorilla gorilla gorilla*  Western Lowland Gorilla | XP_004038593.1 |
| *Heterocephalus glaber*  Naked Mole Rat | XP_012922096.1 |
| *Homo sapiens*  Human | Q9BXR5.2 |
| *Ictidomys tridecemlineatus*  Thirteen Lined Ground Squirrel | XP_005319966.1 |
| *Lipotes vexillifer*  Baiji Dolphin | XP_007449470.1 |
| *Loxodonta Africana*  African Elephant | XP_003411439.1 |
| *Macaca fascicularis*  Crab-eating Macaque | XP_005554730.1 |
| *Macaca mulatta*  Rhesus Macaque | EHH25806.1 |
| *Macaca nemestrina*  Southern Pig-Tailed Macaque | XP_011749692.1 |
| *Mandrillus leucophaeus*  Drill | XP_011838918.1 |
| *Mesocricetus auratus*  Syrian Hampster | XP_005139284.2 |
| *Microcebus murinus*  Grey Mouse Lemur | XP_012609345.1 |
| *Mustela putorius furo*  Domestic Ferret | XP_012917672.1 |
| *Nannospalax galili*  Galilee Blind Mole Rat | XP_008831470.1 |
| *Nomascus leucogenys*  Northern White Cheeked Gibbon | XP_003258625.1 |
| *Ochotona princeps*  American Pika | XP_004579214.1 |
| *Octodon degus*  Degu | XP_004648927.1 |
| *Odobenus rosmarus divergens*  Pacific Walrus | XP_004396223.1 |
| *Orcinus orca*  Killer Whale | XP_004266281.1 |
| *Ornithorhynchus anatinus*  Platypus | XP_001512990.3 |
| *Oryctolagus cuniculus*  European Rabbit | NP_001284430.1 |
| *Otolemur garnettii*  Northern Greater Galago | XP_012667414.1 |
| *Ovis aries*  Domestic Sheep | XP_012034750.1 |
| *Pan paniscus*  Bonobo | XP_008955797.1 |
| *Pan troglodytes*  Chimpanzee | XP_009445754.1 |
| *Panthera tigris altaica*  Siberian Tiger | XP_007098922.1 |
| *Pantholops hodgsonii*  Tibetan Antelope | XP_005961050.1 |
| *Papio Anubis*  Olive Baboon | XP_009204933.1 |
| *Physeter catodon*  Sperm Whale | XP_007102366.1 |
| *Pongo abelii*  Sumatran Orangutan | XP_003776489.1 |
| *Propithecus coquereli*  Coquerel’s Sifaka | XP_012499931.1 |
| *Pteropus Alecto*  Black Flying Fox | XP_006918995.1 |
| *Pteropus vampyrus*  Greater Flying Fox | XP_011363982.1 |
| *Rattus norvegicus*  Brown rat | ACN78428.1 |
| *Rhinopithecus roxellana*  Golden Snub-Nosed Monkey | XP_010364113.1 |
| *Saimiri boliviensis boliviensis*  Black Capped Squirrel Monkey | XP_010336491.1 |
| *Sorex araneus*  Common Shrew | XP_004608124.1 |
| *Sus scrofa*  Domestic Pig | AGT79981.1 |
| *Tarsius syrichta*  Philippine Tarsier | XP_008051197.1 |
| *Trichechus manatus latirostris*  West Indian Manatee | XP_004384732.1 |
| *Tursiops truncates*  Bottle Nosed Dolphin | XP_004321065.1 |
| *Ursus maritimus*  Polar Bear | XP_008699612.1 |
| *Vicugna pacos*  Vicuna | XP_006209809.1 |
|  |  |

**Supplementary Data**

**Sequence 1:** Fasta file of the predicted equine TLR 10 gene from this study.

>ENSECAT00000022185

IQELDIKTFEFNKELRYLDLSYNRLKIVTWYSLTGLRHLDLSFNDFDTMPISEETGNMSHLEILGLSGAKIQKSDFQKIAHLHLNTVFLGLRTLSHYEEGSLPILNTTKLHIVLPMNTNFWVLLRDGIKTSKILEMTNVDGKSQFASYETQQNLILENAKTSILLLNKVDLLWDDLLLIFQFVWHTSVEYFHIQNVTFGGKVYLDHNSFNYSNTVMRAIKLEHVHYRVFYIPQERVYLLFTKMGIENLTISDAQMPHMVFPTCPSKFQYLNFANNILTDDLFKRSIHLPHLKTLILKGNKLETLSLVSCFANNTSLKHLDLSQNLLQHENDENCWWPETLITMNLSSNKFADSVFRCLPQSIQILDLNNNKIQTVPKEMIHLKSLRELNLAFNFLTDLPGCSHFRRLSVLNIEMNLILSPSLDFFQSCQEVKTLNAGRNPFRCTCELRDFIQLEKYSEGMMVGWSDSYICEYPLSLKGTQLKDVHLPELSCNTALLIVTIVVVMLILGMAATFCCLHFDLPWYLRMLHQWTQTWHRVRKATQGQLKRNVQFHVFISYSERDSAWVKHELIPNLEKEDGSVLICLHEGNFDPGNSITENIINHTEKSYKIILVLSPDFVQSEWYRYELDFTHHSLCHENSNYIILILLEPIPLYCIPTRYPKLKALMEKKAYLEWPKDRRKCRLFWANLRAAIHVNLVETRETSELQTFIELNEESQGSAISLIRTDCL
