# Supplementary figures and images for: RNA expression of TLR10 in normal equine tissues
Source: BMC Res Notes. 2016 Jul 19;9:353. doi: 10.1186/s13104-016-2161-9 (PMC4952062; doi:10.1186/s13104-016-2161-9)

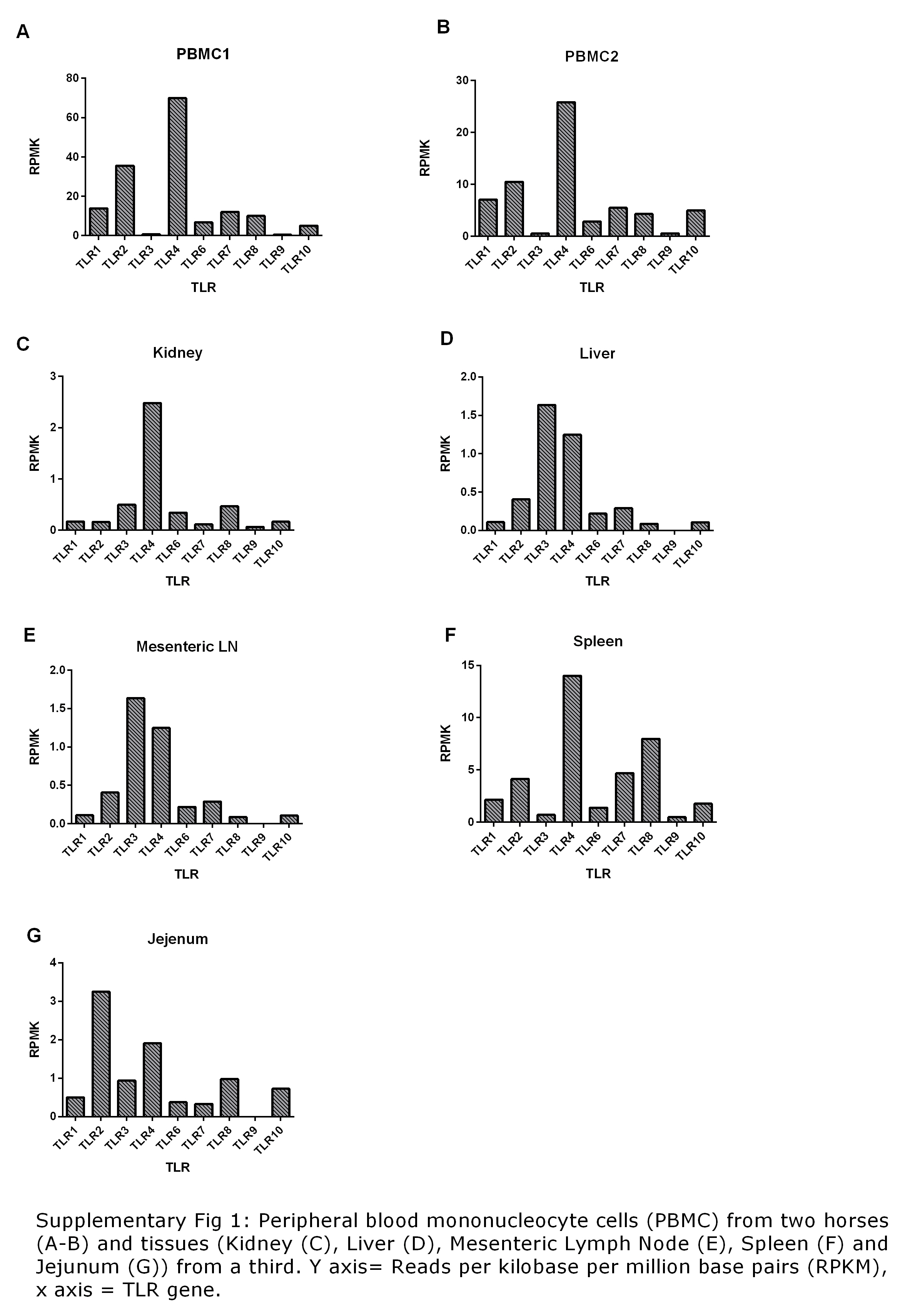

Supplement: Supplementary file 2 — 10.1186/s13104-016-2161-9 TLR 1-4 and 6-10 RNA expression in horse tissues. Peripheral blood mononucleolyte cells (PBMC) from two horses (A–B) and tissues (Kidney (C), Liver (D), Mesenteric Lymph Node (E), Spleen (F) and Jejunum (G)) from a third. Y axis = Reads per kilobase per million base pairs (RPKM), X axis = TLR gene. [file 13104_2016_2161_MOESM2_ESM.jpg]
